# Urban Edge Predators: Wolf Spatial and Temporal Ecology at the Wildland–Urban Interface in Mongolia

**DOI:** 10.3390/biology14091292

**Published:** 2025-09-18

**Authors:** Jeff Dolphin, Maria Vittoria Mazzamuto, Gantulga Gankhuyag, Delgerchimeg Davaasuren, Bayaraa Munkhtsog, Ulam-Urnukh Bayanmunkh, Gansukh Sukhchuluun, John L. Koprowski

**Affiliations:** 1 Haub School of the Environment and Natural Resources, University of Wyoming, Laramie, WY 82072, USA; jeffreyc.dolphin@uninsubria.it (J.D.); jkoprows@uwyo.edu (J.L.K.); 2 Department of Life Sciences and Systems Biology, University of Turin, 10123 Turin, Italy; 3 Institute of Biology, Mongolian Academy of Sciences, Peace Avenue 54B, Ulaanbaatar 13330, Mongolia; gantulga_g@mas.ac.mn (G.G.); delgerchimegd@mas.ac.mn (D.D.); bayaraa_m@mas.ac.mn (B.M.); ulamurnukh_b@mas.ac.mn (U.-U.B.); sukhchuluung@mas.ac.mn (G.S.); 4 School of Natural Resources and the Environment, University of Arizona, Tucson, AZ 85721, USA

**Keywords:** *Canis lupus*, stray dogs, prey availability, livestock, Bogd Khan Mountain, camera traps, human-wildlife interactions, urbanization, activity, abundance

## Abstract

**Simple Summary:**

Mongolia is home to the Bogd Khan Mountain Strictly Protected Area, a UNESCO biosphere reserve and one of the oldest protected areas in the world. This forested mountain is a sky island surrounded by steppe habitat and the rapidly growing capital city of Ulaanbaatar to the north, which has encroached on the protected area since the democratic revolution in 1991. Wolves are the top predators, but they face threats from a growing urban area, livestock grazing, and free-ranging dogs. Using camera traps, we determined the wolf abundance and activity patterns within the protected area. We also modeled wolf activity patterns and examined whether they overlapped with those of people, dogs, livestock, and natural prey. Our results showed wolves avoided humans and livestock and had high spatial and temporal overlap with native ungulates. Wolf abundance was also not determined by the proximity to urban areas. These results will help the protected area administration monitor wolves and other wildlife, as well as identify areas of concern due to the impact of free-ranging dogs.

**Abstract:**

Mongolia’s rapidly expanding capital is encroaching on Bogd Khan Mountain, a UNESCO Biosphere Reserve and the oldest protected area in Eurasia. Gray wolves (*Canis lupus*) in this wildland–urban interface are locally near-threatened due to hunting, local beliefs, and human–wildlife conflict. In 2022 and 2023, we deployed 72 camera traps (11,539 trap nights) to investigate how wolves respond to overlapping pressures from free-ranging dogs, livestock, and human activity. Using a random habitat-stratified camera design and abundance modeling, we assessed diel activity and spatial co-occurrence. Wolves exhibited nocturnal and crepuscular activity, with the greatest temporal overlap with wild prey (wapiti: ∆^4^ = 0.73; Siberian roe deer: ∆^4^ = 0.79), moderate overlap with dogs (∆^4^ = 0.60) and horses (∆^4^ = 0.68), and minimal overlap with cattle (∆^4^ = 0.40) and people (∆^4^ = 0.43). Mean wolf abundance estimates ranged from λ = 0.91 (CI 95%, 0.05–1.77) in 2022 to λ = 1.52 (CI 95%, 0.44–3.53) in 2023. Wolves were more abundant at higher relative abundance of wild ungulates and in areas with more people. Wolves co-occurred with dogs at 11 sites and were more abundant in areas with a higher number of dogs. Our findings highlight the complex dynamics between wildlife, livestock, and human-associated disturbances at the wildland–urban interface, underscoring the need for integrated management strategies that address both ecological and human dimensions of conservation.

## 1. Introduction

Where urban development transitions into wildlands, a wildland–urban interface zone (WUI) is created [1]. The effect of urban areas on neighboring natural ecosystems starts with habitat modification and progresses to direct and indirect effects of human activities at varying scales [2]. Habitat loss, disease transfer, and human–wildlife conflicts (e.g., road kill, livestock depredation, wildlife nuisance) are potential threats with urban expansion [2,3]. For example, predictions of human–carnivore conflicts in the upper midwestern United States showed that livestock depredations occurred more regularly in marginal wolf habitat, and human health–safety concerns and non-hound dog depredations tended to occur in areas with low housing density adjacent to large wildland areas [4]. Therefore, WUIs become a unique social-ecological zone where it is necessary to integrate spatial and non-spatial factors influencing human–wildlife interactions within areas of co-occurrence and analyze WUIs as a continuous surface of multiple overlapping risks [4,5]. Most of the research on WUIs has been focused on wildfire risk, but less is known about how animal communities can be affected in terms of movement, species composition, and interspecific interactions [5].

The gray wolf (*Canis lupus*) in Mongolia has lived for centuries along a human traditional nomadic or semi-nomadic way of life, from the desert and steppe ecosystems to forests and high elevation mountains [6] in what today is the second lowest human population density in the world. However, the developing Mongolian economy has led to a dramatic increase in livestock, mineral extraction, and rapid urbanization, as well as the abandonment of nomadic lifestyles [7]. Wolves were heavily hunted during Mongolia’s communist era, but with the change in the political landscape in 1990, agricultural collectives were dismantled, and herds were distributed as the private property of nomads [1]. As people could now procure more wealth from having larger personal herds, the country saw a dramatic increase in livestock on the landscape from 26 million in 1991 to 64 million in 2024 [8]. Meanwhile, the rapidly expanding capital city of Ulaanbaatar has increased in population by 173% since 1991, thus creating a large wildland–urban interface zone with the UNESCO Biosphere Reserve of Bogd Khan Mountain, a sky island surrounded by a mosaic of different levels of urbanization and steppe. The mountain’s protected status dates back to the 12th century. Officially designated as a Strictly Protected Area by the Mongolian government in 1988, it also achieved international recognition as a UNESCO Biosphere Reserve in 1997 [9]. This makes Bogd Khan Mountain the oldest protected area in Eurasia. The mountain historically hosted 38% of the Mongolian mammal species, including an apex predator, the gray wolf [10]. Herders in the wildland–urban interface of Ulaanbaatar and Bogd Khan Mountain, because of the increase in their livestock numbers, might be at higher risk of wolf depredations and disease transmission. Wolves, on the other hand, might be at higher risk of poaching, habitat loss, and fragmentation related to urbanization, and hybridization and disease transmission from free-ranging dogs. Moreover, the increase in urban population and the encroachment of the city onto the mountain have increased the level of outdoor recreational activities, potentially disturbing and/or displacing wildlife [11].

This study aims to investigate how the Mongolian gray wolf, which has limited knowledge in the changing landscape of its country, responds to various overlapping risks from the wildland–urban interface to natural areas. No previous research has assessed the population of gray wolves near Mongolia’s largest city. We investigated the spatial and temporal responses of wolves to the presence and abundance of free-ranging livestock, humans, and dogs in the landscape. Additionally, we examined whether the distance from urbanized areas influences the abundance of this wolf population on the mountain.

## 2. Materials and Methods

### 2.1. Study Area

**Figure 1:**
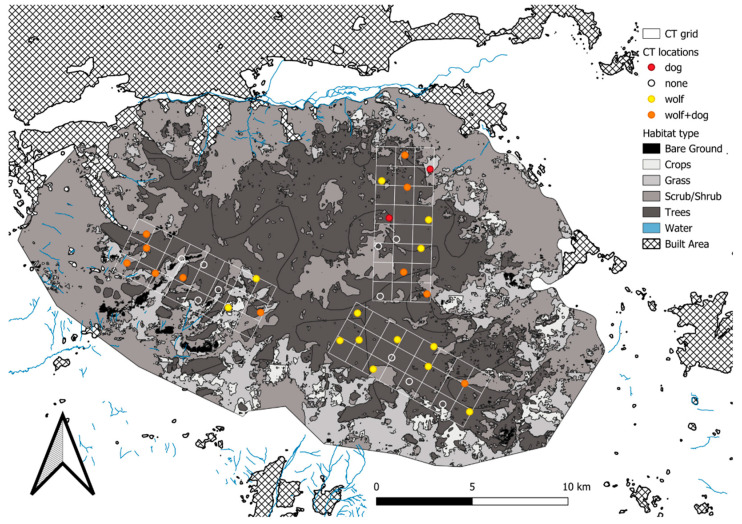
Camera trap locations in Bogd Khan Mountain, Mongolia, in 2022–2023. Colors of the background map show different categories of habitat type (see legend). Circles indicate camera traps: empty circles are those sites where neither wolf nor dog was detected; red circles are sites with dog detections, yellow with wolf detections, and orange sites with detections of both wolf and dog.

Bogd Khan Mountain, situated in north-central Mongolia (Figure 1), is located at the terminus of the Khentii mountain range. Bogd Khan Mountain (2261 m.a.s.l.) is characterized by cold winters (−24–−10 °C) and mild summers (14–17.6 °C) [12]. The mountain’s upper reaches are blanketed by conifer and mixed forests (20,541 ha) dominated by Siberian larch (*Larix sibirica*), Siberian pine (*Pinus sibirica*), Siberian spruce (*Picea obovata*), and birch (*Betula platyphylla* and *B. rotundifolia*) [13]. The forest is encompassed by steppe at lower elevations, which covers the remaining 41,560 ha of the protected area, rendering it a sky island. To the north, Bogd Khan Mountain is facing urban encroachment from the capital city of Ulaanbaatar, which is inhabited by approximately 1.6 million people, as well as urbanization to the south by Zuunmod, a town with a population of about 17,000 (Figure 1). Increasing human population and urban expansion into areas surrounding Bogd Khan Mountain have amplified human disturbance, reducing the forest, grassland and water, and expanding areas of bare soil [14]. Activities such as road construction, building development, outdoor recreation (e.g., hiking and biking), and utilization of natural resources like pine seeds and mushrooms have heightened human presence and placed greater pressure on this mountain ecosystem [11].

### 2.2. Camera Trap Design

We used camera traps to record wolves on the mountain, as well as prey species and potential anthropogenic disturbance (i.e., outdoor recreation, livestock grazing, free-ranging dogs). We placed 72 camera traps in three grids with dimensions 3 × 8 km (24 km^2^) rectangles with 1 × 1 km grid cells (Figure 1). The cameras (Core Low Glow Trail Camera, Bushnell, Overland Park, KS, USA) were positioned at an average elevation of 1862.53 ± 157.31 m (mean ± SD; range 1556–2169 m) using a random stratified habitat design, with one survey site every two km^2^ (Figure 1). We secured cameras to a suitable tree within 5 m of the random location, about 40 cm above the ground. In non-forested habitats (shrubs, grass, crops), we installed camera traps at the edge of the forest facing the open habitat at 10 survey sites. Each survey site had two camera traps placed 100 m apart and aimed in opposite directions to increase the detection probability [15]. The photos collected by the two camera traps at each survey site were considered as a single site, resulting in a total of 36 survey sites. The cameras were programmed to take two photos and one 10-sec video when triggered, with a 30-sec delay between successive trigger events. Cameras were operational at survey sites from April to August in 2022 and 2023. No lure or bait was used. We had 6040 camera trap nights in 2022 and 5499 camera trap nights in 2023. Photos were categorized using the CPW Photo Warehouse access database program version 4.3 [16].

### 2.3. Activity

We examined the diel activity patterns and level of overlap between wolves and two prey species, Siberian roe deer (*Capreolus pygargus*) and wapiti (*Cervus canadensis xanthopygus*), as well as humans and domestic animals (cattle, horses, and dogs). We excluded photos of the same species taken within 30 min at the same location to increase detection independence [17,18]. To assess overlap in the diel activity of coexisting species, we computed the temporal overlap coefficient ∆ [18], which represents the shared area under the two kernel probability densities of the daily activity of two species (∆ = 0 indicates no overlap, ∆ = 1 indicates complete overlap [18,19,20]). We utilized the R package camtrapR version 2.3.0 [21] to calculate the overlap coefficient between the density functions of the daily activity using the estimator Δ^4^ [18]. We converted temporal data from camera traps to radians and employed the Mardia–Watson–Wheeler test of homogeneity (W) to evaluate the hypothesis that the timing of diel activity did not differ for pairs of species [22].

### 2.4. Abundance Index

We conducted a spatial analysis of habitat variables using QGIS software version 3.22.0 (QGIS Development Team 2022, QGIS Geographic Information System, Open Source Geospatial Foundation Project). We generated a 500 m radius circular buffer around each camera and obtained ecological variables associated with land cover type (i.e., forested, grassland, urban, bare ground, and farmland) derived using ESRI 2020 Land Cover imagery. We estimated trends in wolf abundance across Bogd Khan Mountain over two years, in 2022 and 2023. We accomplished this by stacking the data with a one-week detection matrix and using single-season occupancy modeling. Initially, we used the classic MacKenzie [23] occupancy model, which addresses the issue of imperfect detection by employing a repeat visit sampling design. However, our global model for wolf occupancy indicated unexplained variability in detection probability, leading to a poor fit (ĉ = 2.62, *p* = 0.04) as determined by the goodness of fit tests by MacKenzie and Bailey [24]. This overdispersion is consistent with unmodeled heterogeneity in detection probability across sites, a known limitation of standard occupancy models when abundance is not explicitly considered. To address this, we used the Royle and Nichols (RN) [25] model to estimate an index of abundance. This model extends the MacKenzie et al. [23] framework by linking detection probability to latent site-level abundance. This approach not only provides an index of abundance but also accounts for heterogeneity in detection that can otherwise bias occupancy estimates and reduce model fit. In our case, the RN model substantially improved model performance and revealed no evidence of lack of fit (ĉ = 0.92, *p* = 0.44).

Since data were collected over two years (2022, 2023) with limited spatial replication, and our focus was on modeling abundance rather than estimating colonization or extinction dynamics, we “stacked” the data where each site-year was combined and treated as a single site. To address non-independence due to temporal replication across years, we included year as a fixed effect on both abundance and detection probability in all models [26]. To explain the variation in abundance, we examined covariates that may influence the wolf population (Table 1). These covariates included habitat characteristics (forest area, elevation, distance from urban areas), prey availability (relative abundance index of Siberian roe deer and wapiti), and anthropogenic disturbance (relative abundance index of livestock, people, and the number of unique dogs detected by cameras). Relative abundance index (number of species detections/number of trap nights) was calculated with a time resolution of 24 h. We considered all possible combinations of one occasion-level covariate (human detection) and the single site-level covariate (forested/non-forested habitat) on detection, as well as all additive combinations of covariates up to three additive terms. To avoid multicollinearity, we assessed pairwise correlations among covariates using Pearson’s correlation coefficient. Elevation and distance to urban areas were moderately correlated (r = 0.61). Given the relatively low number of wolf detections, and since our primary research objective was to assess the influence of urban areas on wolf abundance, we adopted a conservative approach and retained only distance to urban areas in the models. We tested 40 models with additive combinations of covariates up to four additive terms, one of which was always *year* (Appendix A). Before estimating the model coefficients, we standardized the covariates by subtracting the covariate mean from each site value and dividing the difference by the covariate standard deviation. We tested for spatial correlation between detections using Moran’s I test [27]. We conducted model selection based on AIC and averaged the top models with ∆ AIC ≤ 2 (Table A1).

## 3. Results

**Figure 2:**
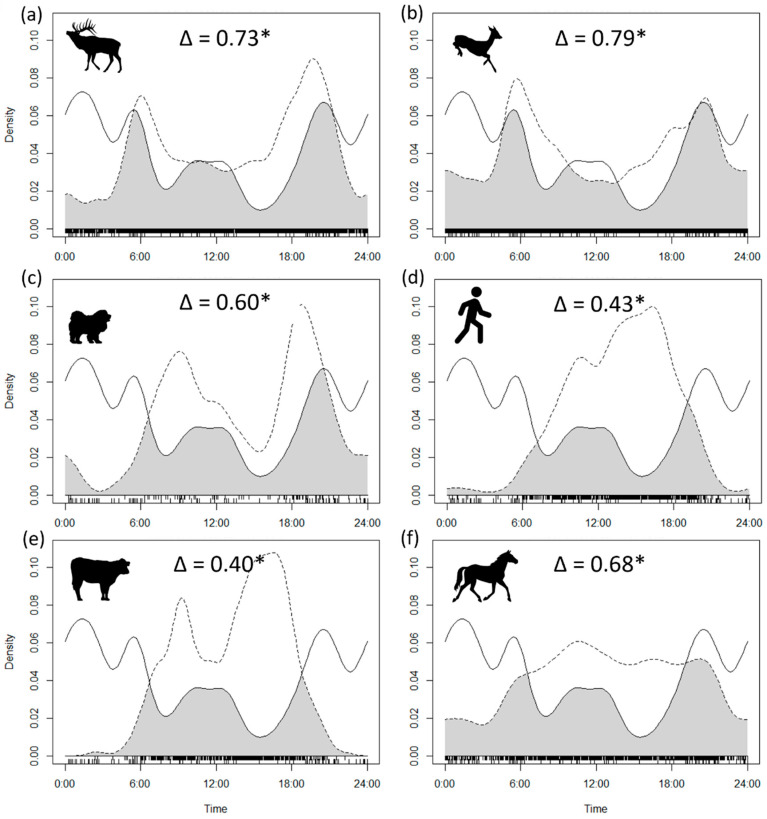
The diel activity patterns of wolves (solid line) and other species (dashed line) in Bogd Khan Mountain, Mongolia. (**a**) Diel activity pattern of red deer and wolves; (**b**) diel activity pattern of roe deer and wolves; (**c**) diel activity pattern of dogs and wolves; (**d**) diel activity pattern of humans and wolves; (**e**) diel activity pattern of cows and wolves; (**f**) diel activity patterns of horses and wolves. The coefficient of overlap (∆) measures the minimum area under the Kernel curve of the probability of activity density estimates, which is represented by the gray area in each graph. The presence of asterisks indicates the significance of the Mardia–Watson–Wheeler test of homogeneity for the hours of activity for pairs of species.

Wolves were active at all hours, with three peaks in activity observed between 2000–0600 h, at dusk, halfway through the night, and dawn. A minor peak was also recorded between 1100 h and 1300 h (Figure 2a–f). Both wapiti and Siberian roe deer were crepuscular (Figure 2a,b). Dogs were diurnal and exhibited a bimodal activity pattern with peaks in the morning and late afternoon (Figure 2c). People were primarily active during the day, with a peak in activity in the afternoon (Figure 2d). Livestock was predominantly active throughout the day, with cattle showing reduced activity during midday (Figure 2e) and horses being active from dawn to the early night hours (Figure 2f). Wolf activity density was lower at higher human and cattle activity. Wolf activity highly overlapped with Siberian roe deer and wapiti (Δ ≥ 0.70), moderately overlapped with dogs and horses (Δ ≥ 0.50), and minimally overlapped with people and cattle (Δ < 0.50). All differences in activity patterns between pairs of species were significant (*p* < 0.0001) (Figure 2).

**Figure 3:**
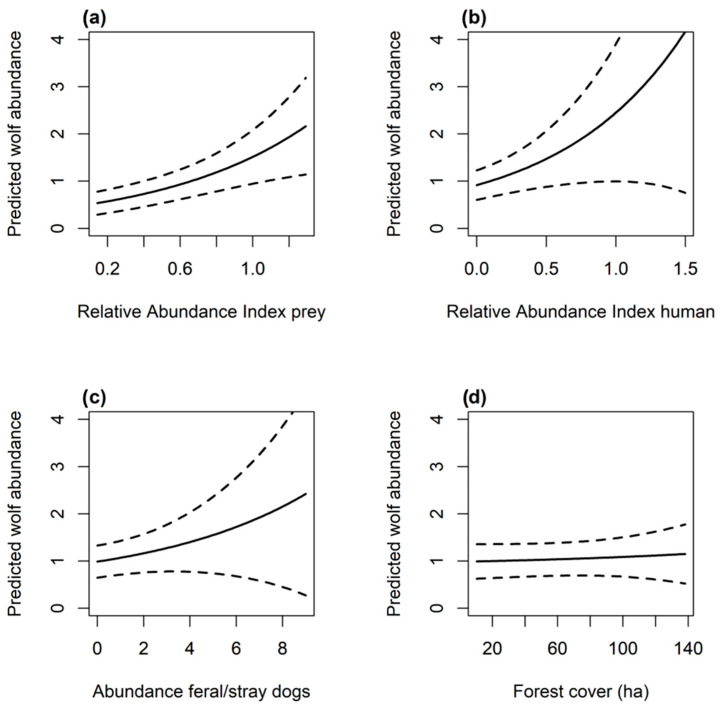
Predictions from the best model λ(year + dog + RAI_Prey + RAI_Human + Forest), *p*(year) describing the abundance (solid line, 95% CI as dashed line) of the gray wolf in Bogd Khan Mountain, Mongolia. (**a**) Predicted abundance of wolves to relative abundance index of humans; (**b**) predicted abundance to relative abundance of prey; (**c**) predicted abundance of wolves to abundance of dogs; (**d**) predicted abundance of wolves to area of forest. For the definition of model covariates, see Table 1.

**Figure A1:**
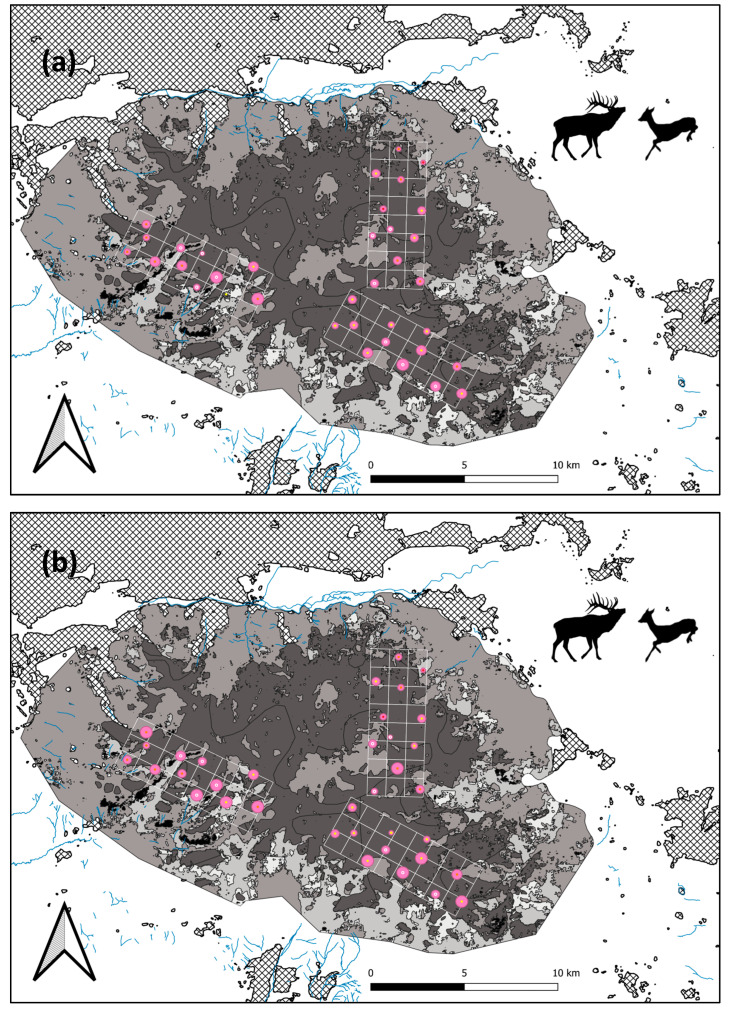
Relative Abundance Index (RAI) of wolf prey (wapiti and Siberian roe deer) in Bogd Khan Mountain, Mongolia, in 2022 (**a**) and 2023 (**b**). Pink circles represent, with their size, the values of RAI divided into fixed intervals. Colored dots within the pink circles indicate camera traps: empty circles are those sites where neither wolf nor dog was detected; red dots are sites with dog detections, yellow with wolf detections, and orange sites with detections of both wolf and dog.

**Figure A2:**
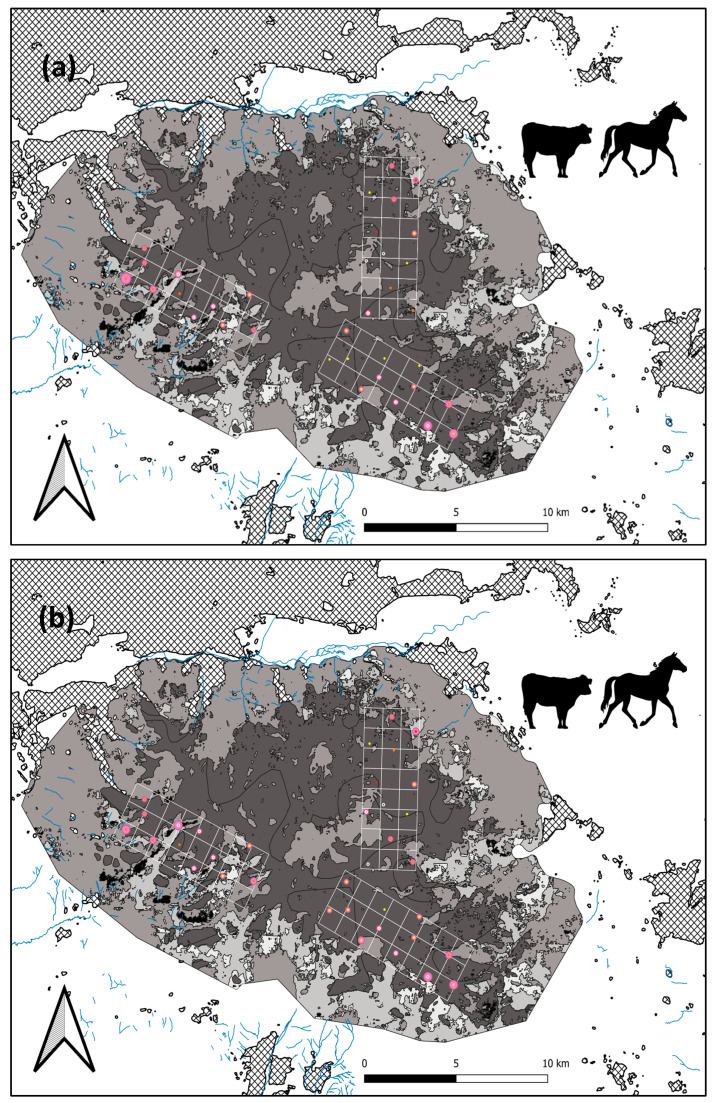
Relative Abundance Index of livestock (cattle and horse) in Bogd Khan Mountain, Mongolia, in 2022 (**a**) and 2023 (**b**). Pink circles represent, with their size, the values of RAI divided into fixed intervals. Colored dots within the pink circles indicate camera traps: empty circles are those sites where neither wolf nor dog was detected; red dots are sites with dog detections, yellow with wolf detections, and orange sites with detections of both wolf and dog.

**Figure A3:**
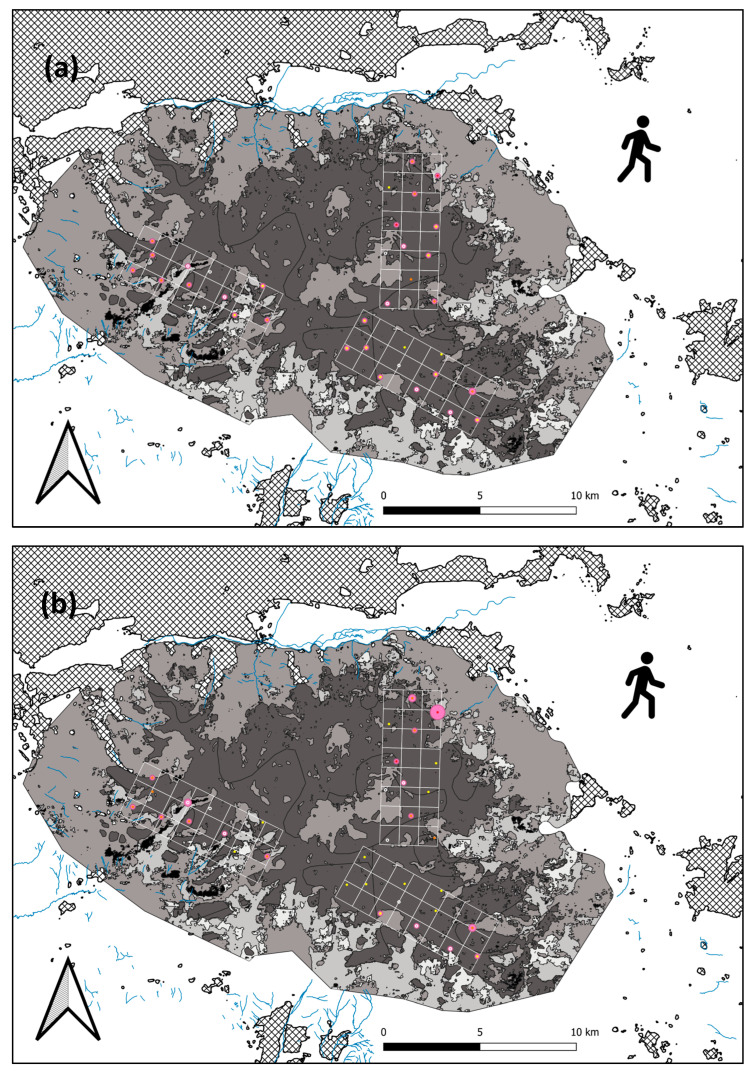
Relative Abundance Index of people in Bogd Khan Mountain, Mongolia, in 2022 (**a**) and 2023 (**b**). Pink circles represent, with their size, the values of RAI divided into fixed intervals. Colored dots within the pink circles indicate camera traps: empty circles are those sites where neither wolf nor dog was detected; red dots are sites with dog detections, yellow with wolf detections, and orange sites with detections of both wolf and dog.

Wolves were detected at 19 sites in 2022 (naïve occupancy = 0.53) and 17 sites in 2023 (naïve occupancy = 0.47) (Figure 3). Most of the detections (n = 75) were of a single wolf, eight detections showed a pair, and only one showed pups. The detections did not exhibit any spatial correlation (Moran’s I observed = −0.12, expected = −0.07, SD = 0.09, *p* = 0.57). Dogs were detected at 6 sites in 2022 (naïve occupancy = 0.17) and 9 sites in 2023 (naïve occupancy = 0.25). Thanks to distinct markings, patterns, or pelage, we identified the number of unique stray and feral dogs detected by cameras at each site. In 2022 on average (mean ± SD) we detected 0.50 ± 1.40 different dogs per site (range 0–7), and in 2023 0.69 ± 1.71 dogs (range 0–9). Wapiti were detected at 35 survey stations in 2022 (naïve occupancy = 0.97) and 34 in 2023 (naïve occupancy = 0.94). Siberian roe deer were detected at all survey stations in 2022 and 2023 (naïve occupancy = 1) [28]. On average, the relative abundance index of prey (wapiti and roe deer) was 0.742 ± 0.334 in 2022 (Figure A1a) and 0.627 ± 0.294 in 2023 (Figure A1b). Horse and cattle were detected at 27 survey stations in 2022 (naïve occupancy = 0.75) and 23 in 2023 (naïve occupancy = 0.64). Goats and sheep were detected only once at one site in 2022 and were not included in the analysis. On average, the relative abundance index of livestock was 0.170 ± 0.227 in 2022 (Figure A2a) and 0.119 ± 0.199 in 2023 (Figure A2b). People were detected at 32 sites in 2022 (RAI = 0.075 ± 0.130; Figure A3a) and 28 in 2023 (RAI = 0.195 ± 0.334; Figure A3b).

The top detection model only included the covariate *year*. The averaged model describing the abundance of wolves in Bogd Khan Mountain was λ(year + dog + RAI_Prey + RAI_Human + Forest), *p*(year). Mean abundance estimates ranged from λ = 0.91 (CI 95%, 0.05–1.77) in 2022 to λ = 1.52 (CI 95%, 0.44–3.53) in 2023. The detection probability was *p* = 0.08 (CI 95%, 0.02–0.14) in 2022 and *p* = 0.06 (CI 95%, 0.01–0.13) in 2023. The model suggested that the RAI of prey (0.39 ± 0.18, z = 2.19, *p* = 0.03) and humans (0.29 ± 0.12, z = 2.49, *p* = 0.01) were associated with a higher predicted abundance of wolves (Figure 3a,b). Wolf’s abundance was higher at higher abundance of stray dogs (0.23 ± 0.12, z = 2.02, *p* = 0.04) (Figure 3c). Year (0.46 ± 0.73, z = 0.63, *p* = 0.53) and area of forest (0.17 ± 0.18, z = 0.97, *p* = 0.33) (Figure 3d) did not affect the predicted abundance of wolves.

## 4. Discussion

The wolf activity patterns observed in Bogd Khan Mountain highlight the ecological flexibility of wolves and the importance of natural prey behavior in shaping predator activity. Wolves adjust their activity patterns and spatial distribution in response to prey availability. Moreover, their moderate overlap with domestic species, low overlap with humans, and higher abundance where people were also more present suggest that they also navigate and adapt to a landscape of anthropogenic disturbance. The impact of stray dogs on wolf abundance suggests a complex interaction that warrants further investigation, as dogs can both compete with and serve as prey for wolves [29,30,31].

Wolf activity patterns vary across other studies, which have attributed these variations to factors such as human activity, breeding status, or prey availability [32,33,34,35,36,37]. However, the high variability of wolf activity patterns is likely a result of their ability to adapt to various environmental conditions [31,38]. Wolves in Bogd Khan Mountain were mainly active at night, with three bouts of activity at dusk, halfway through the night, and dawn. This nocturnal and crepuscular activity pattern mirrors findings from other regions, such as Poland [37,39], the Alps [40], and the Mediterranean [41]. This is thought to maximize hunting success, as many prey species are similarly crepuscular, a pattern also seen in North American wolves hunting deer and moose in Minnesota [36]. On Bogd Khan Mountain, wolves had the highest degree of activity overlap with wapiti and Siberian roe deer, which are crepuscular species. Siberian roe deer have also been the top selected prey animal for wolves in other parts of Mongolia, followed by wapiti [42]. Spatiotemporal overlap is usually high between apex predators and their main prey [43,44,45,46,47,48]. Earlier reports from Bogd Khan Mountain suggested horses (61%) and goats (31%) were the predominant wolf prey, driven by the scarcity of wild ungulates due to poaching [49]. During the two years we conducted this study, we detected only one man carrying a rifle in the spring and summer, and both wapiti and Siberian roe deer are now widely distributed on the mountain. Despite the cathemeral behavior of horses, the activity overlap between wolves and horses was lower than the one between wolves and potential natural prey, possibly indicating the recovery of wild prey and lower predation pressure on livestock, despite recent estimates reporting approximately 5.5 million livestock in the three provinces surrounding the protected area [8]. Cattle had a very limited presence in the diet of Bogd Khan wolves in a study from 2004–2005 [49], in line with the low activity overlap we recorded between cattle and wolves. Moreover, previous studies have noted that wolves often demonstrate temporal partitioning with human-associated species to reduce conflict and competition [32,33,50].

Human activity in our study area was largely diurnal and while wolves were also active during the daytime, they concentrated their activity during nocturnal and crepuscular hours, allowing temporal avoidance from people. In Poland, in heavily forested areas, wolves did not alter their activity in response to human presence [34]. In contrast, in areas of Europe with lower forest cover and higher human densities, such as Italy and Spain, wolves have markedly avoided diurnal activity, likely in response to higher human densities [32,33]. Although human relative abundance was not correlated to prey abundance, people’s activity can alter the behavior of prey species, making them more vulnerable to wolf predation, which in turn increases wolf visitation. In the same study area, it was shown that people had a moderate overlap with wapiti and Siberian roe deer, and that roe deer’s abundance decreased with increasing human abundance [28]. In Greece, predators and prey increased their nocturnal activity at sites of high human disturbance and became more stressed or less vigilant during the evening and night when wolves were active, making them easier for wolves to hunt [51]. This is likely the case for Siberian roe deer and wolves on Bogd Khan Mountain which similarly had high temporal overlap with each other in our study, as well as in similar studies in Mongolia and Greece [42,50,52]. Free-ranging and feral dogs have been shown to display bimodal activity patterns, being active at various times of the day and night. In protected areas, dogs were more active during daylight hours, where they were more dependent on human-provided food resources, which could be the case for dogs in the Bogd Khan mountain area, as they too are more active during the day and avoid being out at night [53].

Wolf abundance increased with the increase in natural prey, whereas the abundance of livestock was not a factor in explaining wolf abundance. This is indicative that wolves are predominantly selecting for natural prey, similar to other areas in Mongolia [42]. Wapiti and Siberian roe deer are widely distributed across most habitats on the mountain [28], and this, paired with the ecological flexibility of wolves, might also explain why the extension of forested habitat did not drive the abundance of wolves. The reduction in temporal overlap between wolves and cattle and the abundance of wolves being mainly driven by natural prey might indicate a lower risk of livestock depredation. This may also be the result of traditional herding practices used for centuries, such as pasture rotations, guard dogs, and sometimes lethal methods to prevent recurring depredations [54,55,56,57]. The abundance of wolves on Bogd Khan Mountain was higher in sites highly visited by people. We included human presence as a covariate to test for possible disturbance effects, and while we did not anticipate a positive association, this pattern is ecologically interpretable. Wolves may benefit indirectly from human activity because prey species often adjust their behavior to avoid people, creating temporal or spatial opportunities that wolves can exploit. In addition, wolves can also benefit from the trails created and maintained by humans for hiking and biking. These trails may serve as efficient travel corridors for wolves, allowing them to move more easily through the landscape [58]. Wolves may be drawn to areas with higher human activity because the trails provide convenient paths for hunting, patrolling territory, or moving between key resources [59].

We observed moderate activity overlap between wolves and dogs, suggesting some shared temporal niche, specifically at dusk. Furthermore, predicted wolf abundance was positively associated with dog abundance. Since wolves and dogs can interbreed and produce viable hybrid offspring, this moderate temporal overlap and the higher chance of encounter at higher abundance of both species can result in hybrid wolf–dog litters being produced [60]. Wolves can prey on dogs [29,30], but the moderate activity overlap between the two species would suggest that wolves are not selecting dogs as a food source [17]. Wolf naïve occupancy and predicted abundance were higher than dog abundance, despite their abundance increasing together in the surveyed sites. This could be attributed to the similarity of the trophic niche that wolves and dogs exhibit. For example, in Italy, both feral dogs and wolves selected wild boar and roe deer (*Capreolus capreolus*) as their primary prey species [61]. In Bogd Khan Mountain dogs are not restricted to or are more common at the edge of the protected area, probably also helped by the existence of a vast array of human and wildlife trails crisscrossing the protected area [62]. Currently, stray dogs are controlled through lethal methods once a month by the city and protected area management, and in 2024, from January to July, 168 dogs were lethally removed (DD pers. comm.).

We did not detect a clear relationship between wolf abundance and distance to urban areas, which may be due to the complex and context-dependent ways wolves interact with human-modified environments. While urban areas typically pose risks such as persecution, vehicle collisions, competition with free-ranging dogs, and disturbance from noise and light, wolves may tolerate or even benefit from proximity to these areas under specific conditions. Wolves persist in human-dominated landscapes when natural habitat patches are nearby, which serve as functional refugia that buffer anthropogenic pressures and support their foraging and movement needs [63,64]. Such habitat heterogeneity may allow wolves to exploit urban edges while minimizing exposure to threats. Moreover, wolves display high ecological plasticity and flexible strategies when coping with human presence [65]. Some individuals may use areas closer to humans for feeding opportunities, especially sub-adults or dispersing individuals [66], while others avoid them altogether [64,65,67,68]. The wide range of individual-specific tolerance and risk-taking in human-modified habitats may obscure any consistent population-level response to urban proximity [63].

## 5. Conclusions

Our findings demonstrate the ecological adaptability of wolves in a human-modified landscape, highlighting their ability to adjust activity patterns in response to prey behavior and anthropogenic disturbance. Despite ongoing urban expansion and the presence of free-ranging dogs, wolves in Bogd Khan Mountain seem to continue to rely primarily on wild ungulates, suggesting a degree of resilience in this changing ecosystem. However, further genetic investigations should assess the potential and degree of hybridization between wolves and dogs as a conservation threat to this population. Persistence of this population will depend on sustaining natural prey populations, mitigating potential impacts from stray dogs, and managing human access and disturbance. These insights contribute to a broader understanding of canid persistence in changing environments and inform strategies for promoting coexistence in protected and transitional landscapes.

## Figures and Tables

**Table 1:** Covariates utilized to model the detection (*p*) and abundance (*λ*) of wolves in Bogd Khan Strictly Protected Area, Mongolia.

Model Component	Covariate	Description
Detection (*p*)	*habitat*	Habitat in which the camera was set up (forest = 0, other habitats = 1)
	*human*	Human presence/absence on each occasion (absence = 0, presence = 1)
	*year*	Year of camera trap survey
Abundance (*λ*)	*forest*	Area of forested habitat (m^2^)
	*elevation*	Elevation of the location of the camera trap
	*urban_dist*	Straight-line distance from camera trap site to the nearest neighboring urban area (m)
	*RAI_prey*	Relative abundance index of Siberian roe deer and wapiti
	*RAI_livestock*	Relative abundance index of cattle and horses
	*RAI_human*	Relative abundance index of people
	*dog*	Abundance of stray or feral dogs
	*year*	Year of camera trap survey

Name of covariates in italic as reported in the text.

## Data Availability

The data presented in this study are available on request from the corresponding author because the data are part of an ongoing study on the mammalian community of the Strictly Protected Area of Bogd Khan Mountain.

## Appendix A

**Table A1:** Models tested to describe the abundance of gray wolf in Bogd Khan Mountain, Mongolia, using the Royle and Nichols (2003) model and subsequent model selection. Detection was kept constant *p*(year). In italics are the most supported models with ∆ AIC ≤ 2.

Model#	Abundance Formula	AIC	∆ AIC	AIC Weight	Cumulative Weight
*model19*	*year + dog + RAI_Prey + RAI_Human*	*434.65*	*0*	*0.16*	*0.16*
*model40*	*year + RAI_Prey + RAI_Human*	*435.47*	*0.81*	*0.10*	*0.26*
*model10*	*year + forest + RAI_Prey + RAI_Human*	*436.55*	*1.89*	*0.06*	*0.32*
*model36*	*year + dog + RAI_Prey*	*436.58*	*1.92*	*0.06*	*0.38*
model16	year + dist_urban + RAI_Prey + RAI_Human	436.81	2.16	0.05	0.44
model13	year + dist_urban + dog + RAI_Human	437.09	2.43	0.05	0.48
model12	year + dist_urban + dog + RAI_Prey	437.45	2.8	0.04	0.52
model6	year + forest + dog + RAI_Prey	437.72	3.07	0.03	0.56
model7	year + forest + dog + RAI_Human	437.92	3.26	0.03	0.59
model37	year + dog + RAI_Human	437.95	3.3	0.03	0.62
model17	year + dog + RAI_Livestock + RAI_Prey	438.15	3.49	0.03	0.64
model25	year + dog	438.21	3.56	0.03	0.67
model39	year + RAI_Livestock + RAI_Human	438.27	3.61	0.03	0.7
model24	year + RAI_Human	438.38	3.72	0.02	0.72
model23	year + RAI_Prey	438.41	3.76	0.02	0.75
model9	year + forest + RAI_Livestock + RAI_Human	438.53	3.88	0.02	0.77
model38	year + RAI_Livestock + RAI_Prey	438.79	4.14	0.02	0.79
model22	year + RAI_Livestock	439.1	4.44	0.02	0.81
model18	year + dog + RAI_Livestock + RAI_Human	439.12	4.47	0.02	0.82
model31	year + dist_urban + dog	439.19	4.53	0.02	0.84
model27	year + forest + dog	439.42	4.77	0.01	0.85
model35	year + dog + RAI_Livestock	439.53	4.88	0.01	0.87
model15	year + dist_urban + RAI_Livestock + RAI_Human	439.62	4.97	0.01	0.88
model30	year + forest + RAI_Human	439.8	5.15	0.01	0.89
model34	year + dist_urban + RAI_Human	439.81	5.15	0.01	0.9
model5	year + forest + dog + RAI_Livestock	440.17	5.52	0.01	0.91
model29	year + forest + RAI_Prey	440.38	5.72	0.01	0.92
model33	year + dist_urban + RAI_Prey	440.41	5.75	0.01	0.93
model8	year + forest + RAI_Livestock + RAI_Prey	440.47	5.82	0.01	0.94
model28	year + forest + RAI_Livestock	440.62	5.96	0.01	0.95
model11	year + dist_urban + dog + RAI_Livestock	440.66	6	0.01	0.96
model14	year + dist_urban + RAI_Livestock + RAI_Prey	440.79	6.14	0.01	0.96
model1	year + forest + dist_urban + dog	440.86	6.21	0.01	0.97
model32	year + dist_urban + RAI_Livestock	441.07	6.42	0.01	0.98
model21	year + dist_urban	441.56	6.91	0.01	0.99
model20	year + forest	441.56	6.9	0.01	0.98
model4	year + forest + dist_urban + RAI_Human	441.57	6.91	0.01	0.99
model3	year + forest + dist_urban + RAI_Prey	442.35	7.69	0.00	1
model2	year + forest + dist_urban + RAI_Livestock	442.54	7.89	0.00	1
model26	year + forest + dist_urban	443.55	8.9	0.00	1
